# Epigenetic basis of diabetic vasculopathy

**DOI:** 10.3389/fendo.2022.989844

**Published:** 2022-12-09

**Authors:** Theja Bhamidipati, Manishekhar Kumar, Sumit S. Verma, Sujit K. Mohanty, Sedat Kacar, Diamond Reese, Michelle M. Martinez, Malgorzata M. Kamocka, Kenneth W. Dunn, Chandan K. Sen, Kanhaiya Singh

**Affiliations:** ^1^ Department of Vascular Surgery, Jefferson-Einstein Medical Center, Philadelphia, PA, United States; ^2^ Department of Surgery, Indiana Center for Regenerative Medicine and Engineering, Indiana University School of Medicine, Indianapolis, IN, United States; ^3^ Division of Nephrology, Indiana University School of Medicine, Indianapolis, IN, United States

**Keywords:** vasculopathy, epigenetics, intravital 2-photon microscopy, type 2 diabetes mellitus, microRNA

## Abstract

Type 2 diabetes mellitus (T2DM) causes peripheral vascular disease because of which several blood-borne factors, including vital nutrients fail to reach the affected tissue. Tissue epigenome is sensitive to chronic hyperglycemia and is known to cause pathogenesis of micro- and macrovascular complications. These vascular complications of T2DM may perpetuate the onset of organ dysfunction. The burden of diabetes is primarily because of a wide range of complications of which nonhealing diabetic ulcers represent a major component. Thus, it is imperative that current research help recognize more effective methods for the diagnosis and management of early vascular injuries. This review addresses the significance of epigenetic processes such as DNA methylation and histone modifications in the evolution of macrovascular and microvascular complications of T2DM.

## Introduction

Ubiquitous across the human body, the vascular organ system comprises the arterial, venous, and lymphatic vessels that perfuse the rest of the living organism. However, due to its widespread location, the vasculature is highly susceptible to injury. At the cellular level, the surrounding environment through which vascular structures seed is integral toward maintaining continuous blood supply. Even the slightest alteration to the extra-vascular space can cause a cascade of pathological consequences from ischemia to neuropathy. Vasculopathy is a broad, all-encompassing term used to describe vascular abnormalities ranging from metabolic derangements to embolic/thrombophilic and functional disorders (1). This term is confused with ‘Vasculitis’ which refers to a more specific inflammatory process dealing with the arterial and venous walls (2). Vasculitis commonly has skin manifestations and is more closely tied to clinical syndromes. Although both terms are used in the nomenclature of vascular pathology, they differ in terms of vessel size and location, and status of inflammation. The nomenclature is largely nonspecific but denotes the vast domain of pathology that can affect the vasculature. For this review, the term vasculopathy will be used to encompass all vasculature-related pathology.

The global impact of vasculopathy cannot be understated (3). Nearly every chronic disease creates derangements in the extracellular space which impacts vascular health. The development of vasculopathy is widespread and represents a large loss of life-years across the world (3). When referring to clinical vasculopathy, diseases such as Kawasaki Disease, Takayasu’s Arteritis, and Giant Cell Arteritis account for the most common cause of acquired cardiac disease in children, elderly Japanese and elderly Europeans, respectively (4). On the venous end of the gamut, chronic venous disease accounts for nearly 2% of the healthcare allocation in western countries (5). More so in the sickest patients such as those immunocompromised or post-transplantation, vasculopathy is the leading cause of transplanted graft loss at a one-year time mark (6). Infectious agents such as Varicella Zoster are also major vascular modifiers and before the advent of the vaccine, 95% of young individuals were infected (7–9).

In the multitude of types of vascular diseases, including the aforementioned vascular diseases, the most prevalent vascular disease in terms of health impact is type 2 Diabetes Mellitus (T2DM)-associated microvasculopathy and macrovasculopathy. It is one of the leading causes of mortality and morbidity in the US with nearly 50% of individuals dying from some form of cardiovascular disease (10). The vascular complications for diabetes span the length of the body similarly remain the number one cause of mortality (11). With the ever-enlarging breadth of literature associated with vasculopathy, there is a clear and present need for a comprehensive understanding of the causes, effects, and potential mechanisms for the development of vascular disease processes. Recent literature (12–24) demonstrates a key role for epigenetic mechanisms such as DNA methylation and histone modification from long standing hyperglycemia in the complex interplay between genes and the environment in diabetic tissue. Promoter DNA methylation induced gene silencing is the most extensive epigenetic modification reported in diabetic vasculopathy (22, 25–28). Such hyperglycemia-induced gene promoter hypermethylation is long known to contribute to a “metabolic memory” that results in vascular dysfunction in diabetes even after achieving glycemic control (15, 23, 29). However, this view has been modified substantially to include small non-coding RNAs or microRNAs (miRNAs) and large intergenic non-coding RNAs as additional epigenetic components (23). In this review, the epigenetic mechanisms associated with pathogenesis of diabetic vasculopathy will be discussed.

## Vascular physiology and pathology

The Vascular system is the organ system comprising of blood vessels connecting the heart to other organs and tissues throughout the body. The blood vessels are thematically split into an arterial side (30) which supplies tissue with oxygenated blood from the heart and a venous side which returns unoxygenated blood to the heart for pulmonary oxygenation. The third group of vessels includes lymphatics and capillaries which manage lymph fluid and extracellular fluid. However, for the point of this review, the focus will be given to arterial and venous vasculature with a primary emphasis on the arteries.

### Pathophysiology of the vascular system

The structure of veins and arteries is a scale of millimeters and smaller. Thus, under circumstances of injury or pathology, there are systemic derangements in the vessels that can occur across the body simultaneously affecting these structures. The breadth of pathophysiology in vascular disease is impressive. Broadly, it can be broken down into aneurysmal disease and occlusive disease (31). The aneurysmal disease is a classification for the development of aneurysms or pathological enlargement of a vessel. This enlargement can affect any vessel but the most notable are aortic aneurysms which carry a high mortality. Underlying the enlargement, however, is a molecular process causing the weakening of the arterial wall. Likewise, the occlusive disease can be broadly attributed to atherosclerotic processes and the development of plaque burden in these vessels. This plaque can then not only alter the blood flow dynamics but can even become entirely occlusive and prevent distal perfusion. Once again there is a complex molecular cascade underpinning the development of atherosclerosis.

### Vasculopathy and vasculitides

Another description of vascular pathology can be termed vasculopathy. While the difference is largely linguistic, it is important to differentiate between these two owing to the difference in clinical implications. Vascular pathology is often referred to as “vasculopathy” which is more appropriate for the occlusive and aneurysmal diseases referred to earlier, vasculitis is important for their existing clinical syndromes. Broadly speaking, the definition of vasculitides is the infiltration of inflammatory cells into the vessel wall causing loss of structural integrity. Furthermore, the 2012 International Chapel Hill Consensus Conference organizes vasculitides based on vessel size, organ dysfunction, and known etiology (32, 33). The most common and studied vasculitides are outlined in **Table 1**. These conditions are more symptomatically outlined and clinically correlated than the term vasculopathy. In nearly all cases of vascular pathology ranging from vasculitides to atherosclerosis, the role of the endothelial layer has been heavily implicated (34).

**Table 1:** Vasculitides Organized based on Vessel Size as well as syndromic presentations. Information drawn from 2012 International Chapel Hill Consensus Conference^13,14.^.

Large Vessel Vasculitis	Medium Vessel Vasculitis	Small Vessel Vasculitis	Variable Vessel Vasculitis	Vasculitis Associated with Systemic Diseases
Takayasu Arteritis	Polyarteritis Nodosa	ANCA Associated Vasculitis Microscopic Polyangiitis Wegener Granulomatosis Churg-Strauss Syndrome	Behcet’s Disease	Lupus
Giant Cell Arteritis	Kawasaki Disease	Immune Complex Vasculitis Anti-GMB Disease Cryoglobulinemia Vasculitis IgA Vasculitis Anti-C1q Vasculitis	Cogan Syndrome	Rheumatoid Arthritis
				Sarcoid Vasculitis

## Endothelium and endothelial dysfunction

The endothelium layer is a thin layer of cells [named Endothelial Cells (ECs)] lining the innermost layer of the vessel lumen. Initially thought as nothing more than a selectively permeable membrane for nutrient transfer, the role of the endothelium has largely expanded to nearly every aspect of cardiovascular physiology (34). Previous research delineated the role of ECs in angiogenesis, homeostasis, and immune response (35). These cells commonly line the inner layer of vascular organs and act as the direct contact with potential vascular derangements. In this role, they can control vascular tone, leukocyte adhesion, and the tight regulation between pro- and anti-coagulative environments (36). Originally described as a monolayer along with basal lamina cells, ECs have always been implicated in a diverse role operating as much more than a simple inert barrier (36). New literature has shown that this endocrine organ has affector and effector properties allowing for maintenance of homeostasis across the body (37). More so, the homeostatic mechanisms can further be divided into mechanisms of (i) Coagulation and Thrombolysis, (ii) Leukocyte Interactions, and (iii) Vasoconstrictor/Vasodilator Regulation (37). Importantly, endothelial cells are under the most immediate effect of any alterations to flow dynamics, viscosity, or plaque formation. Thus, any dysfunction in these tightly controlled mechanisms can cause devastating downstream effects across the entire vasculature. Any shift away from the abovementioned role of ECs is largely defined as an instance of endothelial dysfunction. Reduced vasodilation, increased inflammatory markers, and creation of prothrombotic conditions are the hallmark of a dysfunctional endothelial layer (34). In the most severe form, this dysfunction can be lethal resulting in coronary events, kidney failure, diabetes, and formation of free radicals. The cause of endothelial dysfunction is multifactorial and has been attributed to diabetes, smoking, hypertension, and general inactivity (38). Diabetes creates a multifactorial pathology that causes endothelial dysfunction over the course of decades.

## Diabetes and vasculopathy

### Impact

According to the National Diabetes Statistics Report of 2020, 30 million people over the age of 18 live with T2DM, and another 68 million live with the ascribed diagnosis of ‘prediabetes’. However, more than the current incidence, the rising prevalence of the disease is most worrisome. Growing exponentially in younger populations, diabetes is becoming one of the largest losses of life-years in the modern healthcare system. T2DM itself is characterized by derangements in insulin responsiveness and glycemic control. This creates an environment of chronic inflammation and radical oxidization that harms small and large neurovascular bundles which is referred to as diabetic vasculopathy. More so there is an element of atherosclerotic burden that causes increases in plaque development in the lumens of arteries. This same process occurs in coronary vessels and makes coronary artery disease a dominant cause of mortality among diabetics (39). In the peripheral vessels this disease process is referred to as Peripheral Arterial Disease (PAD) and costs over 200 billion USD (40, 41). The most frequent and morbid outcome of PAD is amputation. At 5 years, nearly 50% of amputees die (40, 42).

Diabetic Vasculopathy is an encompassing term used to describe vascular complications associated with systemic hyperglycemia and hypertension seen in diabetes. The hallmarks of diabetic vasculopathy include injury to large vessels such as the aorta and coronary vessels as well as more peripheral injuries such as retinopathy and nephropathy. Coronary vessels susceptible to plaque burden have a much different pathophysiologic profile than small retinal vessels. The environment created by uncontrolled hyperglycemia affects vessels differently depending on their size. Thus, a dichotomy exists where we can explore the effects of diabetes on large vessels (macrovasculopathy) and small vessels (microvasculoapthy).

### Etiology of diabetic vasculopathy

The large impact of diabetic vasculopathy, creates a need to understand the molecular mechanisms of the disease process. Early proposed etiologies of this vasculopathy stem from the treatment of different pharmaceutical therapies and then retroactively applying a cause. For example, the Renin-Angiotensin-Aldosterone System has been implicated in this process due to the beneficial effect of ACE Inhibitors and AT1 receptor antagonists (11). Similarly, the treatment of vasculopathy with endothelin inhibitors implicated the role of hyperplasia as a cause of vasculopathy. These inhibitors have been shown by Marano et al., to decrease luminal hyperplasia in carotid arteries (43). However, a true elucidation of the mechanisms requires a full investigation.

The literature on the mechanisms for diabetic vasculopathy is divided into two types: physical and molecular with the former slowly accumulating over years. Metabolic factors in conjunction with altered hemodynamic flow cause the abnormal release of cytokines and vascular factors (44). Cytokines such as prosclerotic cytokine transforming factor B (TGFβ) are overexpressed in glomeruli and kidneys seen in diabetic nephropathy (45). Likewise, growth factors such as vascular endothelial growth factor (VEGF) and its receptor VEGFR2 were found in increased in the retina (11). The downstream effects of this process cause alterations in blood flow and abnormal remodeling and plaque development causing the most physical changes associated with vasculopathy such as extracellular matrix accumulation and atherosclerosis.

Peripheral tissues rely on much smaller caliber vessels for perfusion. Thus, any change to the diameter of the lumen can drastically change the physiologic profile of the organ in question. For example, the afferent glomeruli vessels can lose vascular tone due to cytokines and inflammatory markers causing increased perfusion throughout the nephron. Downstream this can destroy the glomerular basement membrane and cause podocyte effacement and albuminuria. Alongside this loss of filter, is the expansion of the extracellular matrix from profibrotic signals causing tubulointerstitial fibrosis (46, 47). Together this creates the clinical picture of diabetic nephropathy. Likewise, in retinal tissue increased reactive oxygen species and loss of signaling from platelet-derived growth factor (PDGF) cause increased angiogenic factors like Tie 2 causing vascular proliferation and the prototypical proliferative diabetic retinopathy (46, 47). The three major aspects of diabetic vasculopathy are large vessel atherosclerosis, nephropathy, and retinopathy (46).

**Figure 1:**
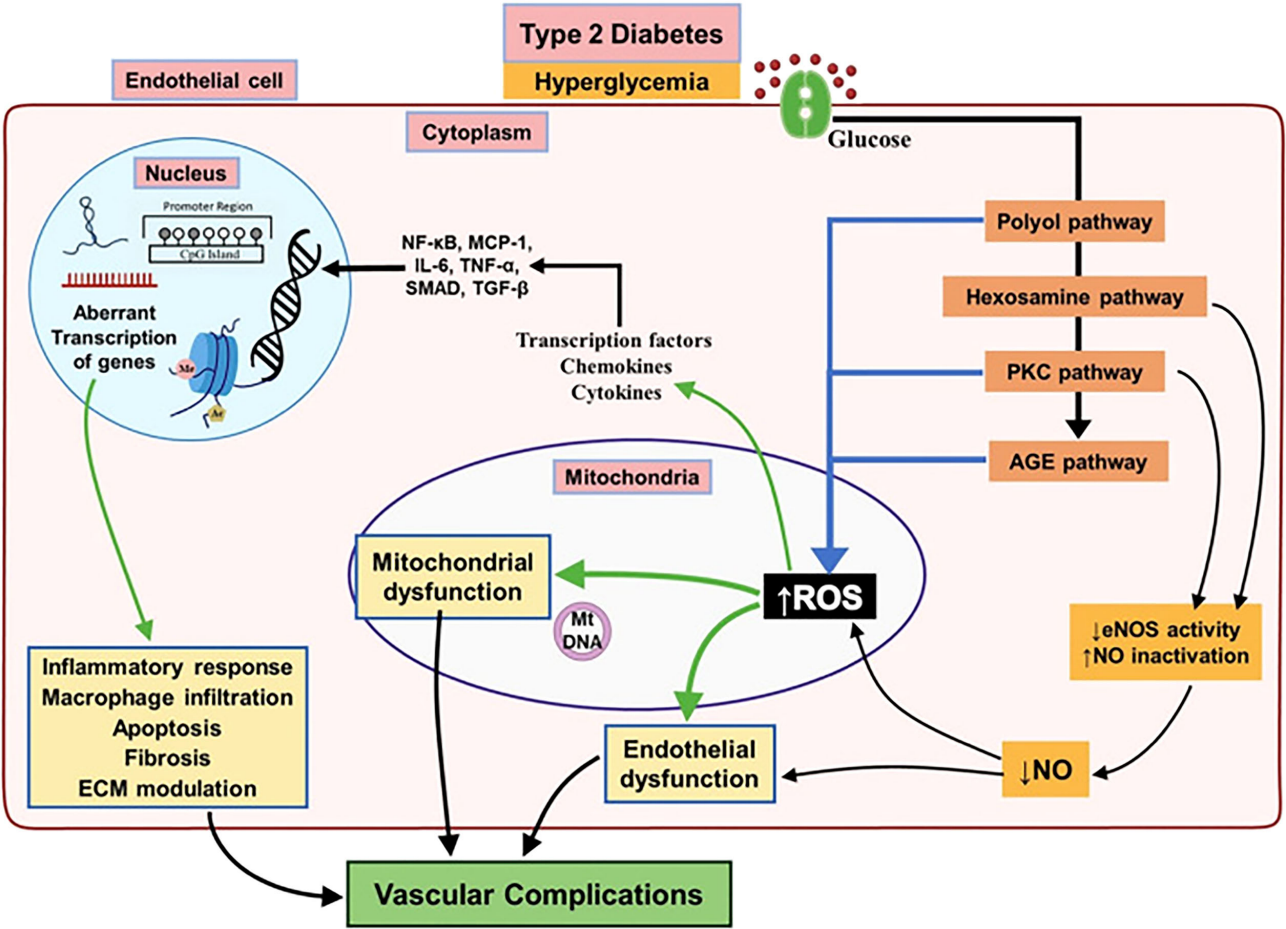
Major biochemical and cellular pathways underlying endothelial dysfunction leading to vascular complications associated with T2DM. Chronic hyperglycemia activates different pathways involved in leading to endothelial dysfunction in T2DM subjects result in decreased activity of eNOS and increased mitochondrial ROS overproduction. This will bring up a pro-inflammatory environment by activation of several mediators which alter the endothelial epigenome and followed by aberrant transcription of genes regulating inflammatory response, macrophage infiltration, apoptosis and fibrosis resulting in diabetic vascular complications. Reproduced with permission from Elsevier. Following original report was credited: Dhawan et al. (51).

As mentioned earlier, the mechanisms for both large and small vessel deterioration are exacerbated by the hyperglycemic environment created by diabetes. Specifically, there are three states that persistent hyperglycemia can cause. Hyperglycemia is being used as the hallmark of uncontrolled insulin resistant/exhausted T2DM (48, 49). Advanced glycation results from the excess sugar present in the blood stream causing sugar to be added to molecules such as lipids or proteins creating advanced glycation end products (AGEs) (50). These AGEs can cause downstream complications where they create cross-links in the basement membrane and downstream cascades resulting in increased reactive oxygen species (50). Together, these may result in impaired vessel permeability and vasculopathy (**Figure 1**). The second metabolic derangement of diabetes is the activation of the Protein Kinase C pathway (PKC) (50). This chemical cascade primarily regulates vascular smooth muscle contractility. In diabetes, the AGEs and reactive oxygen species mentioned earlier prematurely activate the PKC pathway causing vasodilator dysfunction and downstream vasculopathy of both large and small vessels (52). Lastly, the Sorbitol accumulation in the Polyol pathway is the third metabolic derangement of diabetes that can contribute to vasculopathy (52). The polyol pathway is a two-step pathway converting glucose into fructose. An intermediate in this path is sorbitol which is unique because it cannot pass the cell membrane and remains extracellularly creating osmotic stress. In conditions of diabetes such as under excess glucose, more and more sorbitol accumulates causing increased osmotic gradients and oxidative damage to vessels (53). All in all, the elucidated mechanisms are heavily researched and make up the current dogma of diabetes as it relates to vascular dysfunction. However, it is noted that these mechanisms are all chemical cascades of known molecular pathways. There is a lack of a comprehensive review of the models that dictate these mechanisms at an extra-genomic level.

### Diabetic vasculopathy and peripheral neuropathy

Peripheral neuropathy is an encompassing term used to define peripheral nervous system injuries like numbness, tingling, burning, and pain (54). The pathophysiology of the development of peripheral neuropathy is complex and there are many, overlapping etiologies ranging from systemic autoimmune disorders to physical compression (54). However, one of the most common causes of peripheral neuropathy is diabetes accounting for over 50% of the cases (55). This incidence is so high that the World Health Organization has even coined the phrase diabetic peripheral neuropathy (DPN) to account for the large amount of comorbidity (55). The most prominent damages to the nerves in diabetic patients suffering from DPN includes nerve fiber damage, axonal loss, and endoneurial microangiopathy (56, 57). Based on these findings, research from Malik et al. showed a positive correlation between symptoms of clinical neuropathy and microvessel changes (58). This correlation was further investigated by Yagihashi et al. (2010) who showed that the hyperglycemic environment of diabetes did cause microangiopathic damage and downstream neuropathic symptoms (59). Their research showed that hyperglycemia caused derangements in the polyol flux causing a hyperosmolarity of sorbitol in the cytoplasm of neurons and eventual lysis (60, 61).

Additionally, the role of glycated end products such as AGEs in the pathophysiology of DPN has come under investigation. AGE metabolites are highly accumulated in the arteries of diabetic patients (62). However, these end products were also found to be present in the axoplasm and Schwann cells exerting an injurious process on peripheral nerves. Additional signaling pathways such as the Protein Kinase C (PKC) Pathway are also inappropriately activated during diabetic vasculopathy (**Figure 1**). Most notably the PKC pathway has been heavily implicated in the control of nerve function (63). Lastly, oxidative stress and pro-inflammatory states caused by diabetic vasculopathy (from chronic hyperglycemia) increase cellular senescence and enhance the development of DPN (64). Oxidative stress created free radicals which have been shown to decrease nerve conduction velocities (65). Thus, diabetic vasculopathy is a prime causative agent in the development of neuropathic symptoms. The multifaceted etiology mentioned above means that damage to vascular structures during conditions of hyperglycemia does not occur in isolation. Surrounding nerves and axons are also susceptible to diabetic derangements. The investigation into diabetic vasculopathy also yields important information about the role that vascular injury plays in the development of DPN. In this review, we will further explore the epigenetic components that create vascular dysfunction in conditions of diabetes.

## Epigenetics and diabetic vasculopathy

**Figure 2:**
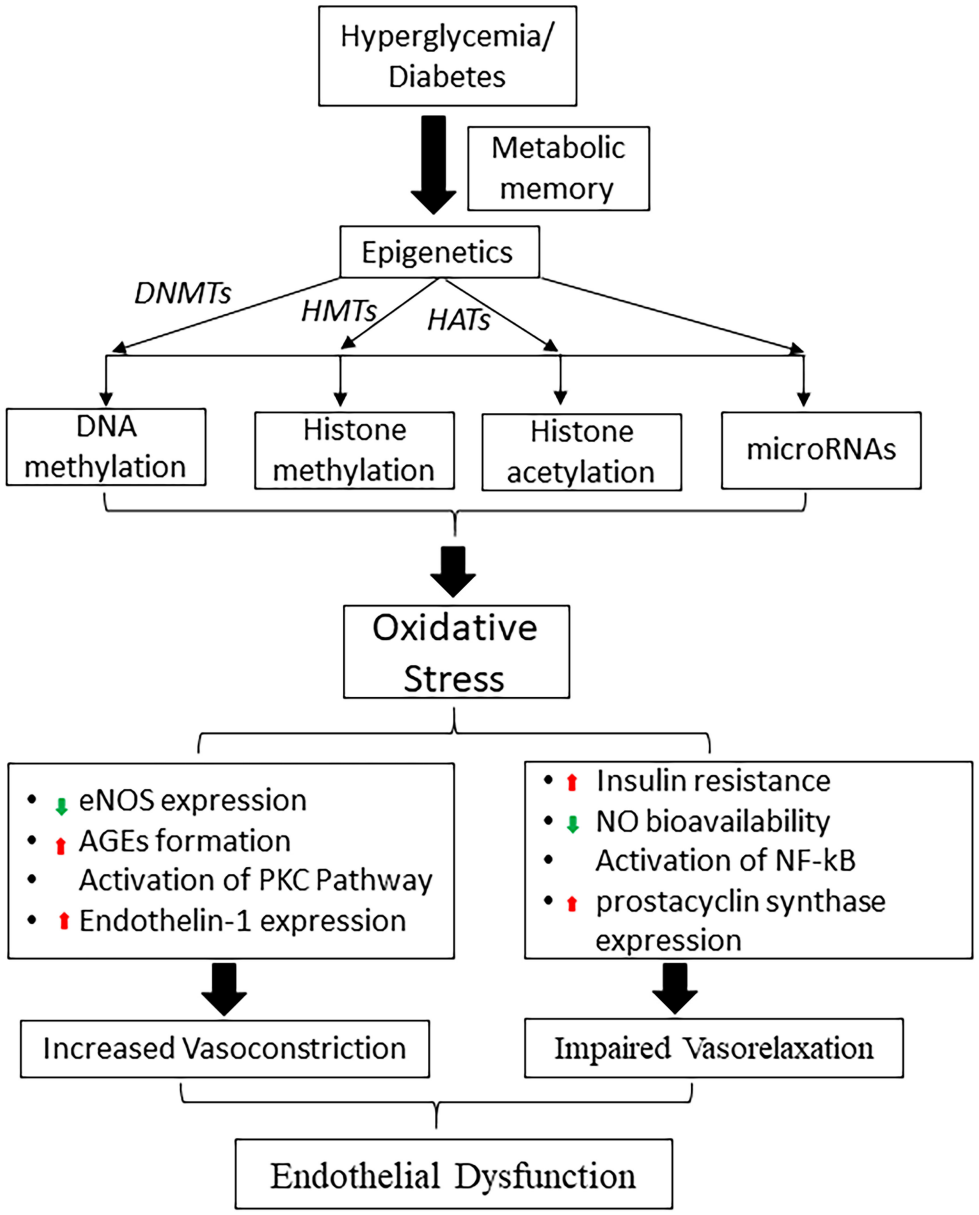
Schematic diagram shows the role of hyperglycemia induced epigenetic mechanisms in metabolic memory and diabetic endothelial dysfunction. DNMTs, DNA methyltransferases; HMTs, histone methyltransferases; HATs, histone acetyltransferases; eNOS, endothelial nitric oxide synthase; NO, nitric oxide, AGE, advanced glycation end products; PKC, protein kinase; NF-κB, nuclear factor-κB.

**Figure 3:**
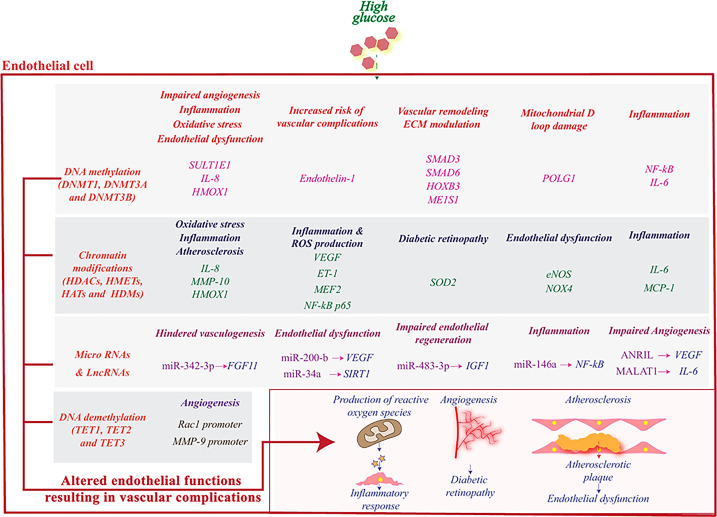
High glucose induces epigenetic reprogramming in endothelial cells and influences genes that are associated with its (dys)functions. DNA methylation and demethylation, histone modifications, miRNAs and lncRNAs regulate the activity of various genes related to angiogenesis and stimulate pathophysiological pathways leading to an inflammatory response and subsequently to vascular complications. Reproduced with permission from Elsevier. Following original report was credited: Dhawan et al. (51 *)*.

Epigenetic modification is a broad-encompassing term that denotes changes in gene transcription that are not due to changes in DNA sequence (66). These modifications mostly regulate the nucleosomal arrangement around DNA and control the gene activation or inactivation. The five prototypical examples to be discussed in this article include 1) DNA methylation, 2) Histone modifications, 3) Chromatin Remodeling 4) microRNAs and 5) long non-coding RNAs (67, 68) [**Figure 2**, reviewed in Singh et al., 2020 (24)]. These different situations point to a unique aspect of genomic regulation in that external factors, outside the domain of DNA sequences, can regulate complex disease processes (69–72). T2DM has a very complex inheritance pattern with an intricate intersection between genetics and environmental factors. This interplay mimics the function of epigenetic modification and heavily implicates a strong epigenetic role in the development of diabetes and diabetic vasculopathy (51, 73) (**Figure 3**).

### Epigenetic mechanisms of diabetic vasculopathy

Under the paradigm of vascular dysfunction mentioned earlier, we can further explore the specific epigenetic modifications that occur in diabetic vasculopathy and how it fits into the micro/macroangiopathic picture. The current literature is diverse as it relates to diabetic vasculopathy. A large all-encompassing study by Chen et al., 2016 (74) examined the role that poor glycemic control has in diabetic microvascular complications. Using the DCCT Trail [Diabetes Control and Complication Trial (1983-1993)] researchers showed that microvascular changes between a strict glycemic group and a control group were significant (75). The control group without glycemic regulation showed increased signs of the angiopathic complications mentioned earlier (retinopathy, nephropathy). Interestingly, sites of DNA methylation were different between the control and glucose-restricted group (76). This study set the stage for the role of epigenetics in diabetes and confirmed the previous studies implicating uncontrolled hyperglycemia in the role of vascular derangement.

Nakatochi and researchers found that in the field of myocardial infarction, of which diabetes is over 40% comorbid) there are three unique sites (77). These unique sites along the DNA were found to have hallmarks of DNA methylation (24, 78, 79). More so correlative analysis among Japanese men showed that DNA methylation at these sites was not only predictive of myocardial infarction but also predictive independent of BMI and lipid levels. This points to the independent regulation of large vessel disease *via* epigenetics. Going further, Bell et al., 2010 (80) found 19 CpG sites with correlations to diabetic nephropathy when compared to the control among 192 Irish patients. Already we see epigenetic regulation at both a macrovascular level as reported by Nakatochi et al., 2017 (77) and at a microvascular level as reported by Bell et al., 2012 (81) across diverse demographic groups and appropriately matched by age and comorbidities.

The field of diabetic retinopathy falls under microvascular complications of diabetes but is more complex due to the heavy collateralization of the retina. Researchers including Berdasco 2017 examined exudative and ischemic damage to retinal vessels (82). Both changes are consistent with the chronic issue of diabetic retinopathy and the development of blindness in uncontrolled diabetes through pro-angiogenic factors (83, 84). In a three-step model, researchers found proliferative retinopathy consistent with exudative and ischemic damage had 46 genes marked by CpG island methylation (85). More so the entire MAP3K1 pathway, which is beyond the scope of this article, was found to be hypomethylated near the promotor. Hypomethylation of regulatory genes is consistent with the dogma of increased gene expression. Increased gene expression of this pathway correlates with increased proliferative retinopathy. More so, Argadh et al., 2015 (85) investigated the comprehensive DNA genome implicated in the disease. The group examined DNA methylation rates at over 300 CpG sites. The findings were consistent not only with an increased level of methylation rates among those with diabetic retinopathy, but the rate of methylation went so far as to work as a predictive algorithm for the severity of the retinopathy.

Only limited studies have examined the role of histone modification and chromatin remodeling in the pathogenesis of diabetes and vascular diseases. Under hyperglycemia, pancreatic islet-specific transcription factor Pdx1 recruits co-activators p300 and the histone methyltransferases (HMTs) SET7/9 to increase histone acetylation and H3K4me2 (23). Such recruitment results in the formation of open chromatin at the insulin promoter and stimulation of insulin production. On the other hand, under low glucose conditions, the same transcription factor, Pdx1, could recruit co-repressors HDAC1/2, leading to inhibition of insulin gene expression (23). Persistent inflammation in diabetic tissue, elevates inflammatory gene expression in endothelial cells through an increase in histone lysine acetylation (86, 87). For example, H3K9/14Ac and histone acetyltransferase (HATs) CBP/p300, play key roles in inflammatory gene expression in diabetic tissue (88). Taken together, these findings suggest that diabetic stimuli can trigger changes in the promoter methylation and chromatin structure that can have long-lasting effects on the expression of target genes.

### Epigenetic basis of non-healing diabetic wounds

One area of chronic complications from T2DM that falls outside of the domain of vasculopathy is the associated ulcers caused by vasculopathy. While not a direct vessel injury, non-healing diabetic ulcers are a direct result of the ischemia associated with arterial insufficiency and decreased perfusion. In particular, the cutaneous mechanism of wound healing in response to injury is unbalanced due to diabetic oxidative stresses and abnormal gene-environment interaction (89–97). ECs dysfunction and accumulation of glycosylated products as described earlier cause damage to the step-wise process of scar formation and healing (68). Several epigenetic mechanisms, particularly DNA methylation and histone modifications have been observed during cutaneous wound healing process (98). Specific examples include the reduction of trimethylation of H3K27 (H3K27me3) in the murine dermal wounds. This was associated with increased expression of H3K27‐specific lysine demethylases Jmjd3 and Utx. In addition to this, components of the polycomb repressive complex 2 (PRC2): Eed, Ezh2 and Suz12, which methylate H3K27, were found to be downregulated during murine wound healing (99). Another gene that remains in tight epigenetic control is eNOS. In physiologic conditions, endothelial cells show constant activation of eNOS *via* a largely hypomethylated promotor region along symmetric strands and across CpG dinucleotides. More so, research from Yan et al., 2010 indicates that the chromatin structure of eNOS is also different among endothelial cells versus nonendothelial cells. In particular, a histone deacetylase (HDAC) inhibitor has even been shown to upregulate eNOS levels (100). Thus, the dysregulation seen in diabetic wounds of the levels of eNOS shows a direct pathway to the role that epigenetic regulation will have in wound healing.

The role of macrophages also comes into question as the dysfunction seen in nonhealing wounds directly relates to the level of a gene called Monocyte Chemoattractant Protein-1 (MCP-1). In streptozotocin-induced diabetic macrophages, the MCP-1 gene was shown to be increased compared to control mice. This increased expression was in direct epigenetic control *via* the MCP-1 promoter region by monomethylation (68). Chromatin remodeling is one of the important epigenetic modifications involved in regulating the transcription of inflammation-associated genes that affect macrophage polarization and other properties essential for successful wound healing (101). Specifically, HAT and histone deacetylase (HDAC) activate inflammatory monocyte differentiation and macrophage phenotype. Alteration of macrophage-related genes by histone modification enzymes correlated with impaired wound healing (102). Further, the ATP-dependent chromatin remodeling (SWI-SNF) complex plays a role in macrophage development. The SWI-SNF interacts with HDAC1 to regulate histone acetyltransferase (H3K27ac) and regulate genes which is important for cell development and differentiation (103).

### Non-coding RNA-based therapeutics in diabetic wound healing

**Figure 4:**
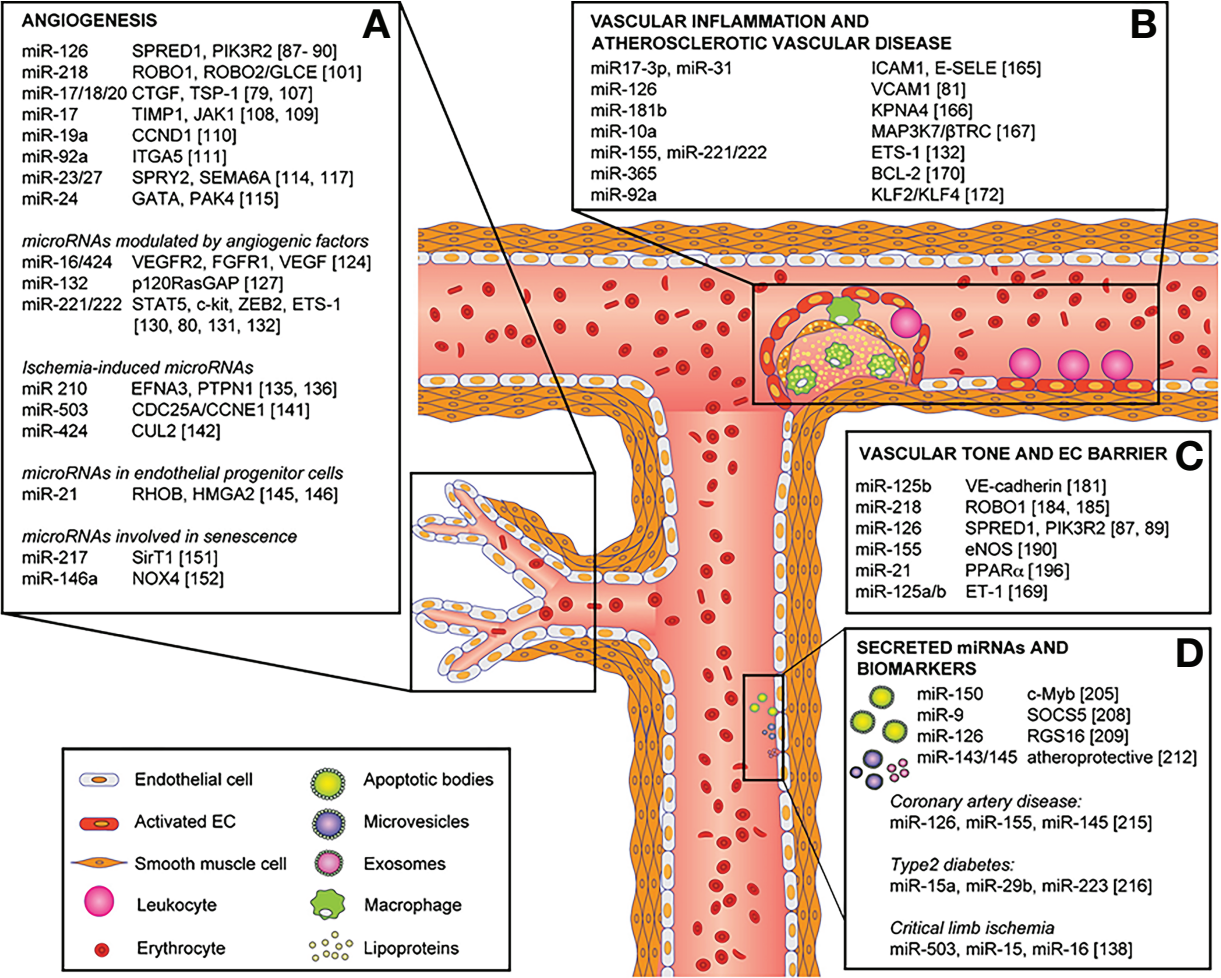
Role of miRNAs in endothelial cell phenotype, functions and vascular disease and their direct targets. MicroRNAs involved in: **(A)** angiogenesis, **(B)** vascular inflammation and atherosclerotic vascular disease, **(C)** vascular tone and endothelial cell barrier, and **(D)** secreted microRNAs and biomarkers. Reproduced under the terms of the Creative Commons CC BY license published by Elsevier. The following original report is credited: Chamorro-Jorganes et al. (105).

MicroRNAs are short single-stranded which instead of being translated into proteins, bind strongly to mRNA affecting gene expression. The therapeutic potential of miRNAs is worth exploring as: i) a single miRNA can act as an amplifier by regulating multiple functionally convergent target genes, ii) miRNAs are stable small biomolecules that can be manipulated with emerging techniques, and iii) they can be delivered precisely in a controlled manner. The therapeutic efficacy can be achieved either by over-expression of specific miRNA or by silencing it. The delivery of potentially therapeutic biomolecules is achieved using viral or non-viral methods for gene therapy using emerging nanotechnology-based approaches (104). Studies have shown deregulation of miRNAs functions is associated with diabetes pathogenesis and complications (**Figure 4**) (105). For example, endothelial cells have been shown to have a specific upregulated miRNA in severe hyperglycemia and an analogous upregulation in the plasma derived from diabetic foot ulcers (105, 106). A further 14 miRNAs were found to have a variable expression in diabetic conditions compared to control mice according to Madhyastha et al., 2011 (107). Specifically, it was found that during diabetic wound healing miR-146b was upregulated nearly 30 times (107). MiR-21 on the other hand was reduced during diabetic wound healing (107). Li et al., 2009 found that treatment with a miR-221 inhibitor lowered levels of miR-221 and improved cell migration under hyperglycemic conditions (108).

MiR-200b has long been implicated in the role of diabetic vasculopathy. Previous literature had shown that miR-200b had a regulatory role in the angiogenesis of diabetic wounds (109). However, there was the question of the regulatory role of miRNA in hyperglycemia. We explored the role that hyperglycemia has on the promotor of miR-200b *via* epigenetic modifications (18). We found that there was noticeable hypomethylation of the promotor region of miR-200b seen in diabetic wound-site endothelial cells in severe hyperglycemia. This work showed the first evidence of the hypomethylation status of miR-200b (18). More so this fact is confirmed by a methyl donor S-adenosyl-L-methionine rescued endothelial function by re-methylating promotor regions. Therefore, manipulation of the expression of specific miRNAs may be an effective therapeutic approach to overcoming diabetes-associated complications (110). The overexpression and downregulation of specific genes regulates wound biology which is chiefly regulated by miRNAs. Unraveling the process of dysregulated miRNAs in skin wound healing will help in developing new targeted therapies. OxymiRs are miRNAs working in response to tissue oxygenation state (111). A wide range of oxymiRs has been studied which are differentially expressed during wound healing (112). Hypoxia-sensitive miRNAs are termed “HypoxymiRs” (113). Chronic non-healing wounds like diabetic foot ulcers, venous ulcers, and pressure ulcers are characterized by ischemia/hypoxia (112). In this regard, miR-203 miR-210 and miR-21 are well-explored hypoxymiRs actively involved in wound healing (114). Similarly, miRNAs play a very crucial role in inflammation control during the healing of the wound (112). miR-155, miR-146a, and miR-132 are some of the miRNAs explored in relation to wound and inflammation (115). For example, miR-155 regulates the expression of proteins involved in the immune response against pathogens which has clinical significance in chronically infected wounds (116). It is also involved in regulating TNFα through other signaling mediators (117, 118). miR-125b (119), miR-31, miR-17-3p (120) and miR-124a (121) are other miRNAs involved in inflammation regulation in wound healing. miRNAs also play a pivotal role in maintaining barrier function during re-epithelialization of skin wounds (112, 122). miR-210-dependent pathways impair ischemic wound re-epithelialization (123). Also, overexpression of miR-1 in skin keratinocytes impairs cell migration ultimately affecting re-epithelialization and skin barrier functions (124–126). miRNAs have a very important role in angiogenic response during wound healing through guiding vascularization (127, 128). An array of miRNAs is involved in the angiogenesis process in different stages - proliferation, migration, and morphogenesis of endothelial cells. A few examples of miRNAs involved in angiogenesis also termed as angiomiRs, include miR-15b, miR-16, miR-20, miR-21, miR-23a and others (112, 129). The above miRNAs can be applied in clinical settings by modulating their expression *via* gene therapy approach. However, the existing challenge is the effectiveness and specificity of delivery to relevant tissues/organs in an active form (130). In an ideal condition, cellular uptake of the delivered miRNA should be high and without endosomal escape (131). Another limitation is that the approach of treatment targeting one miRNA can have undesired off-target effects because of its downstream effects on multiple genes.

Long non-coding RNAs (LncRNAs) are another set of non-coding RNAs that play a significant role in vascular signaling. LncRNA regulates gene expression by regulating chromatin dynamics and transcriptional activities. LncRNAs are prominently deregulated in diseases such as cardiovascular disease, diabetes, and primary open-angle glaucoma (132–134). For example, lncRNA ZEB-AS1 acts as a miR-200b sponge to regulate cell migration, invasion, and proliferation (135). However, these lncRNAs may also contribute to the progression of T2DM disease or other related diabetes-related complications (134). Growing evidence indicates that multiple lncRNAs are involved in diabetic complications, and multiple angiogenic miRNA‐lncRNA pairs relate to wound healing in the maturation phase. During the wound healing process LncRNAs GAS5, IGF2AS, MALAT1, ANRIL, H19, MIAT and lncEGFL7OS are reported to regulate angiogenesis process (136). Additionally, it has been reported that the increased circulating lncRNAs NKILA, NEAT1, MALAT, and MIAT expression in patients with T2DM may influence the degree and severity of disease among patients with T2DM (134). Diabetic wound angiogenesis thus operates under closely regulated epigenetic control.

## Vascular tissue imaging modalities

A rise in the prevalence of diabetes worldwide predicates the significance of incorporating non-invasive imaging modalities in the management of diabetic vascular diseases. Non-invasive imaging of vasculature continues to provide functional parameters for monitoring pathophysiological complications in diabetic patients. Ultrasonography (USG) is a useful imaging modality available to characterize anatomical vasculopathy in non-coronary arteries (137). The USG is also a valuable non-invasive imaging method used to illustrate peripheral vasculopathy contributing to the development of diabetic skin ulcers (138). Furthermore, color Doppler US technology uses a slightly different frequency that enables the measurement of blood flow through blood vessels (18, 139, 140). Pulse wave Doppler velocity measurement is useful in providing relatively precise arterial size measurement which is the foundation for identifying feeder vessels (18, 139, 140). Identifying differential rates of blood flow in the tissue surrounding diabetic ulcers is crucial to preventing further complications that could ultimately lead to amputation (137). Other non-invasive 3D imaging devices like thermography, macrophotography, laser speckle perfusion mapping, and laser Doppler flowmetry modalities are also clinically relevant to the management of vascular diseases (141, 142).

Non-invasive imaging modalities also offer accessible tools to monitor vascular tissue microcirculation in addition to measuring blood flow perfusion as a pathological diagnostic measurement of tissue vasculature. Computed Tomography (CT) is an X-ray-based technique used for characterizing changes in microvascular morphology. Magnetic resonance imaging (MRI) is another method that has been used to evaluate tissue vascular volume, microvascular flow, and permeability of biomarkers for T1DM and T2DM in rodent models (143). Additionally, Hyperspectral imaging has been used to quantify tissue oxygenation in the application of diabetic foot wound care (144). Evaluating a spatial map of oxy- and deoxyhemoglobin in the tissue surrounding diabetic foot ulcers can determine the burden of early medical interventions reducing the potential for amputation. However, the lack of specificity in these imaging modalities makes it hard to differentiate cell types that have a different pathological origin (137). Diagnostic clinical applications of targeted imaging techniques would provide health-care providers with detailed information about vascular processes at the cellular level. To that effect, nano-particles containing multiple biomolecular targets in hybrid imaging techniques on lower mammals create a molecular contrast in activated endothelial cells (145–147). Additionally, researchers have used nuclear labeling techniques in single-photon emission computerized tomography (SPECT) analysis for measuring the localization of leukocytes (148). However, the limited spatial resolution of nuclear imaging can cause poor anatomical localization (137, 148). Intravital Microscopy (IVM) is an imaging technique that offers an improved resolution of vascular tissue in small animals.

### Intravital microscopy in the assessment of diabetic vasculopathy

IVM is a microscopic technique that can track the biological changes, cell function and cellular response in real-time and subcellular level in a live animal. It combines 3-dimensional reality *in situ* with real-time detailed analysis at the subcellular level (149). IVM provides a whole dynamic nature of the live structure of organisms under observation. Among other benefits, IVM (i) is compatible with a great range of labeling methods, (ii) enables time-course dynamic imaging in situ, (iii) does not rule out complete interplay in intact, *in vivo* system, (iv) provides high spatial resolution, (v) even subcellular context at a molecular level can be examined, (vi) quantitative data, (vii) over time analysis decreases the number of animals used in the study (150). All contrast methods used in IVM work well with laser scanning microscopy and any multimodal microscopy.

In the context of healing wounds, the application of the IVM technique can acquire high-resolution images and reveal the composition of the wound, flux of migratory cells and how vascular tissue elements respond to potential drugs and treatments. The combination of 2 Photon-IVM (2P-IVM) and a growing variety of mouse strains with fluorescent reporters have paved the way to evaluate the involvement and influence of particular dermal elements such as hair follicles, glands, vessels and nerves therein during dermal immune responses (151). 2P-IVM can spot collagens in the skin owing to second-harmonic generation (SHG), which facilitates and simplifies trails of cells and invader organisms. Similar to this common fiber visualization, there several agents staining blood (Evans Blue, dextrans), lymphs (anti-LyVE-1) and dermal cells (e.g. CellTracker™ CMTMR, CMTPX dyes) (151).

**Figure 5:**
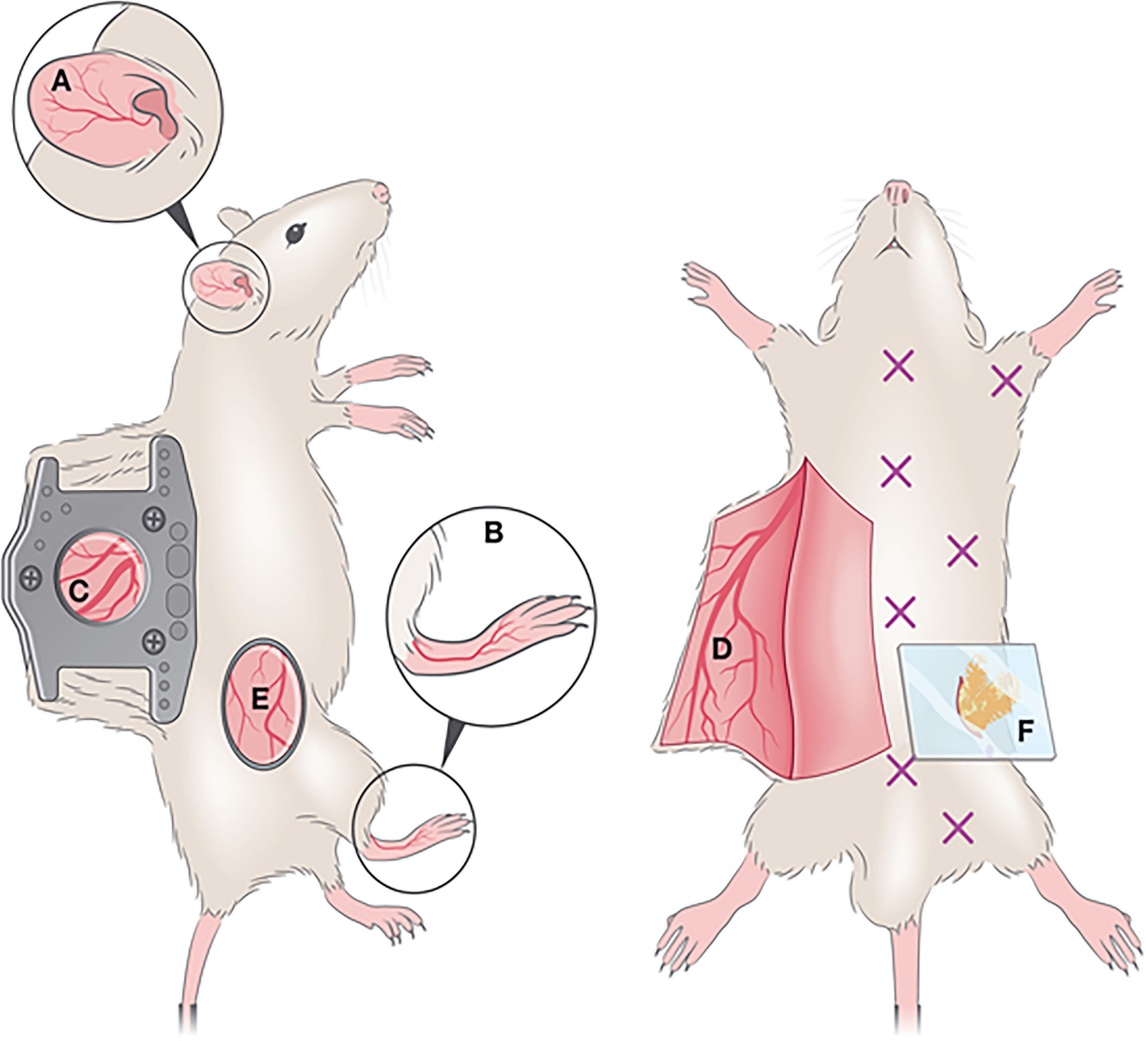
Optical windows and imaging chambers for skin and adipose tissue visualization by IVM. Non-invasive methods include **(A)** ear pinna imaging and **(B)** foot-pad imaging. A semi-invasive method **(C)** includes the dorsal skinfold chamber, which requires the surgical implantation of two titanium or polymer frames that can hold a ring with a glass coverslip through which imaging is performed. An invasive method is the generation of a skin flap **(D)**, whereby a skin flap is generated, exposing a large imaging area. This procedure is invasive and terminal. A less commonly used method for IVM imaging is the skin flank **(E)** which requires the generation of an incision at a dorsolateral location, and either direct imaging or mounting on a stainless-steel disc for stable image acquisition. For adipose tissue imaging, various types of windows exist to visualize various depots (marked by X). To image the perigonadal adipose tissue, a terminal lower abdominal window **(F)** was generated. Reproduced under the terms of the Creative Commons CC BY license published by John Wiley and sons. The following original report is credited: De Niz et al. (152).

In IVM, dorsal ear, flank, footpad and dorsal skin imaging are the most commonly used models (**Figure 5** **)**. Since different skin parts vary in terms of milieu, response, cellular composition, fiber components, nerve and vessel network, etc., researchers select the imaging model as per their particular study or adapt the model to their requirements. Overall, these variabilities can affect skin studies (153). Ear pinnae imaging is a compatible method for infection, injury, allergy and hypersensitivity-related studies (154). According to the requirement of the studies, different skin imaging models are developed. In the case of HSV-1 virus, infecting the epicutaneous part of the skin, a larger surface area is needed than the ear pinnae model presents. As such, the skin flank imaging model started to be employed, a good model to explore viral lesions and responses at the outer dermal layers (151). It links minimal surgery, better reproducibility, and the accession of the lesional part without cauterization. Dorsal and footpad imaging are preferred in invasive and longitudinal studies including microvascular regeneration, wound healing (155), and dermal tumors (156–158), rather than infection-related studies. Dorsal skin imaging is longitudinal and non-invasive imaging and is appropriate for infection-related studies. With a skin-fold dorsal skin chamber model (155), repetitive intravital images can be taken (150).

**Figure 6:**
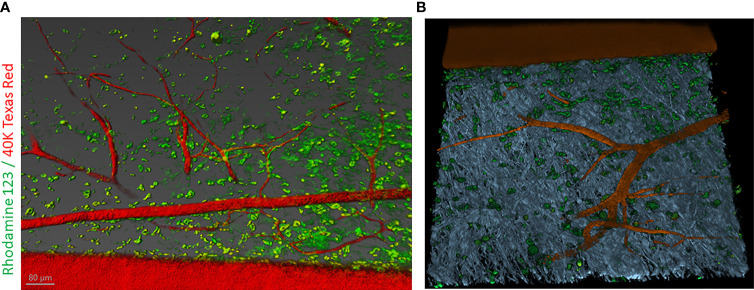
Intravital labeling for two-photon microscopic imaging of adult mouse skin vasculature. **(A)** Texas Red 40 KDa dextran marked the perfused vasculature and Rhodamine-123 marked the cytoplasm of cells present nearby the vasculature containing mitochondria. Scale bar = 80 µm. **(B)** Using Imaris software the 3-dimensional vessel structure was reconstructed from a region of interest (ROI) from panel **(A)** Grey color represent second-harmonic generation (SHG) indicative of collagen in the skin tissue.

In this article, we examined the disposition of a cationic lipophilic fluorescent dye, rhodamine 123 (RH123) used as a mitochondrial-specific stain to measure mitochondrial membrane potential in the IVM (159). In addition, Dextran, Texas Red™, 40,000 MW (40kDA), was used to mark vascular elements in the adult mouse skin. Briefly, the C57BL/6 mouse was anesthetized using 5% isoflurane in an induction chamber for up to 2-3 min. After that, the mouse was mounted to a nose cone with 2-3%. After a surgical level of anesthesia was reached, the mouse was placed on a plastic plate with a heating pad covered with a surgical drape to maintain the body temperature at ~37.5°C. The depth of anesthesia was measured by toe pinch. To visualize vasculature and follow dynamics changes, first, animals were injected with 20mg/ml solution containing 40kDA dextran Texas Red™ dissolved in 1X PBS, then followed with the injection of 20mg/ml solution containing RH123 through the jugular vein. Z-stack images were taken of the same visual field as observed by time-lapse imaging and processed with Imaris software (Oxford Instruments) (**Figure 6**).

At its current stage, IVM has some limitations such as (i) limited depth into the tissue up to a few hundred microns, (ii) clearance of fluorescent dye, (iii) costly labeling by systemic injection that entails high doses of probes, (iv) unknown and hardly estimated marker amount reaching the target, (v) inflexibility to utilize different animal disease models, (vi) mostly based on murine models and (vii) anesthesia duration. These issues need to be addressed and optimized with future improvements during tissue-level analyses of diabetic vasculopathy in a live animal.

## Conclusion

The domain of vascular pathology ranges from acute illness to chronic debilitating disease. T2DM and diabetic vasculopathy has a large breadth of literature surrounding intricate chemical cascades and genomic findings implicating the role of genetics in the disease process. Ranging from large vessel atherosclerosis to small peripheral intimal hyperplasia and endothelial dysfunction, diabetic vasculopathy is a widespread multiorgan pathology that has limited therapeutic intervention. However, there is a lack of comprehensive review of the types of mechanisms by which diabetic vasculopathy is regulated. In specific the regulatory processes of diabetes that occur extrinsically in DNA were not fully explored. This dimension of epigenetics is particularly interesting for diabetes due to the multifaceted transmission of the disease. Diabetes has long been considered not only a genetic disease but a disease that relies on environmental cues for expression. This complex interplay is seen in the inheritance patterns of T2DM. This same dogma applies to the epigenetic regulations that we have examined earlier where epigenetic regulation is analogous to environmental stressors producing a diabetic phenotype. While the molecular role of diabetes has been largely explored with dozens of chemical cascades being outlined, there is still a dearth of literature specifically targeting the regulatory mechanisms of diabetes. This article reviews not only the regulatory mechanisms of diabetes, but also the epigenetic regulations of diabetic vasculopathy. These regulatory processes occur exclusively outside the domain of DNA structure and order and thus are appealing for their potential therapeutic targets.

In particular, the examination of the epigenetic regulations of diabetic vasculopathy has shown that the involved epigenetic regulators have multiple roles. Loss of angiogenesis not only affects diabetic wounds but can also cause limited perfusion, systemic hypertension, and cutaneous wound healing. On the other hand, the increase in angiogenesis seen in diabetic retinopathy works on an opposite theme where excess blood vessel proliferation causes worsening retinopathy. Thus, there is still a role to elucidate the specific role of epigenetic modifications as they change across the body. Similarly, the role of microRNAs has a strong regulatory function, but the function is largely dependent on the environment such as hyperglycemia. Interestingly, the role of epigenetics offers itself as a prime therapy for pharmaceutical intervention. Since these mechanisms operate outside of the genome and do not have downstream cascades, alterations to these methylation proteins or histone modifiers can have a profound impact on the treatment of diabetic vasculopathy. This will of course be built on fully understanding how these regulatory agents work and specifically under what conditions. This may create a situation where therapy is indicated at a certain threshold (such as an HbA1C percent) but is contraindicated at lower or physiologic levels.

## Author contributions

TB, MK, SV, SM, SK, DR, CS and KS wrote the manuscript. SV, SK, KS, MM, MK and KD participated in the intravital microscopy experiment on the mouse skin. All authors contributed to the article and approved the submitted version.
